# Predicting and Explaining Yields with Machine Learning for Carboxylated Azoles and Beyond

**DOI:** 10.1021/acs.jcim.4c02336

**Published:** 2025-02-07

**Authors:** Kerrin Janssen, Jonny Proppe

**Affiliations:** TU Braunschweig, Institute of Physical and Theoretical Chemistry, Gauss Str 17, 38106 Braunschweig, Germany

## Abstract

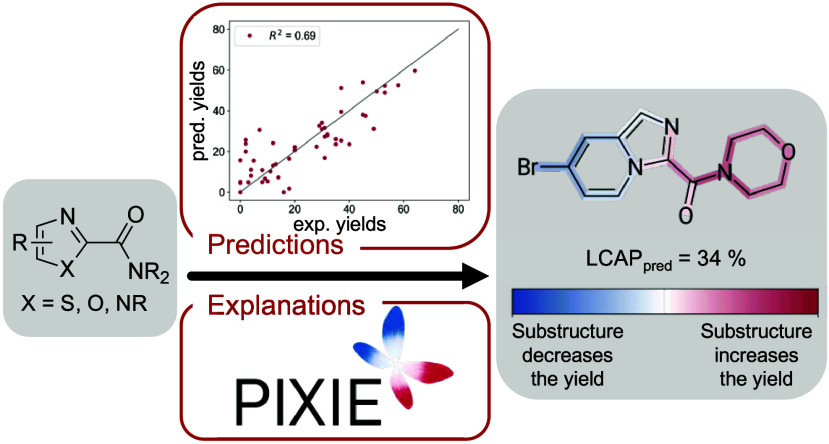

Carbon dioxide (CO_2_) can be transformed into valuable chemical building blocks, including C2-carboxylated 1,3-azoles, which have potential applications in pharmaceuticals, cosmetics, and pesticides. However, only a small fraction of the millions of available 1,3-azoles are carboxylated at the C2 position, highlighting significant opportunities for further research in the synthesis and application of these compounds. In this study, we utilized a supervised machine learning approach to predict reaction yields for a data set of amide-coupled C2-carboxylated 1,3-azoles. To facilitate molecular design, we integrated an interpretable heat-mapping algorithm named PIXIE (Predictive Insights and Xplainability for Informed chemical space Exploration). PIXIE visualizes the influence of molecular substructures on predicted yields by leveraging fingerprint bit importances, providing synthetic chemists with a powerful tool for the rational design of molecules. While heat mapping is an established technique, its integration with a machine-learning model tailored to the chemical space of C2-carboxylated 1,3-azoles represents a significant advancement. This approach not only enables targeted exploration of this underrepresented chemical space, fostering the discovery of new bioactive compounds, but also demonstrates the potential of combining these methods for broader applications in other chemical domains.

## 1 Introduction

While carbon dioxide (CO_2_) is a key driver of climate change,^1−3^ it is also recognized as a versatile carbon source in organic chemistry due to its role as a C1 building block and its abundant availability and production.^4−8^ Consequently, synthesis protocols that transform CO_2_ into valuable molecules are of great interest.^5−7,9−14^ Among these valuable molecules are 1,3-azoles, a prominent subgroup within the broader class of azoles.^15^ 1,3-Azoles and their C2-carboxylated derivatives are already known to be relevant as anticoagulants, herbicides, fungicides, and aroma compounds.^16−18^ C2-carboxylated 1,3-azoles could also enable new applications as prodrugs and propesticides, as well as in treatments for Bartter’s disease or breast cancer.^15^

**Figure 1:**
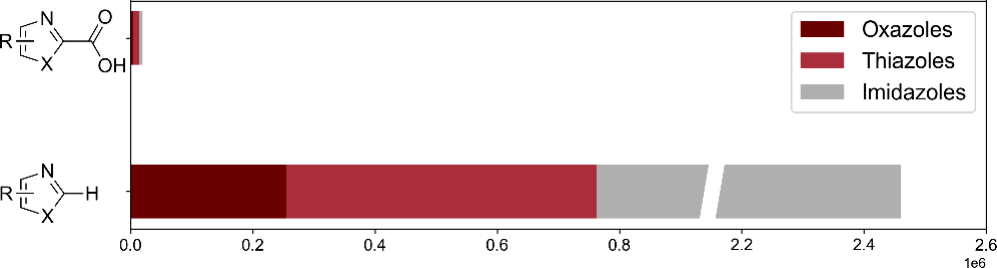
Number (in millions) of commercially available compounds that feature a 1,3-azole substructure (with X = NR, O, S), excluding compounds with a molecular weight exceeding 900 Da, as well as compounds containing metal ions or isotopes.

Despite their advantages, only about 20,000 C2-carboxylated azoles are commercially available, compared to almost 2.5 million C2-unsubstituted 1,3-azoles (CAS SciFinder search in 07/2024; Figure 1).^15,19^ This highlights significant potential for expanding the chemical space of these compounds since access to a broader variety of molecules facilitate the discovery of new bioactive compounds and, therefore, drugs.

**Figure 2:**
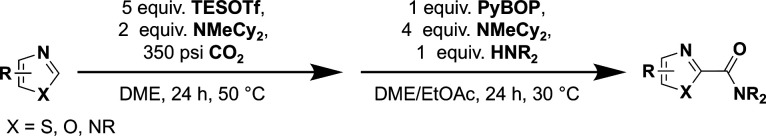
Synthesis protocol developed by Felten et al.^19^

Felten et al. developed a mild, functional-group-tolerant synthesis protocol for the carboxylation of 1,3-azoles, followed by a subsequent amide coupling reaction (Figure 2).^19^ The results of this protocol were reported as liquid chromatography area percent (LCAP) yields. One data set from the study comprises 288 LCAP yields, obtained by combinatorially pairing 24 1,3-azoles (ten thiazoles, nine oxazoles, five imidazoles) with 12 amines, see Figure 2. The LCAP values of this combinatorial set range from 0 to 78%.

**Figure 3:**
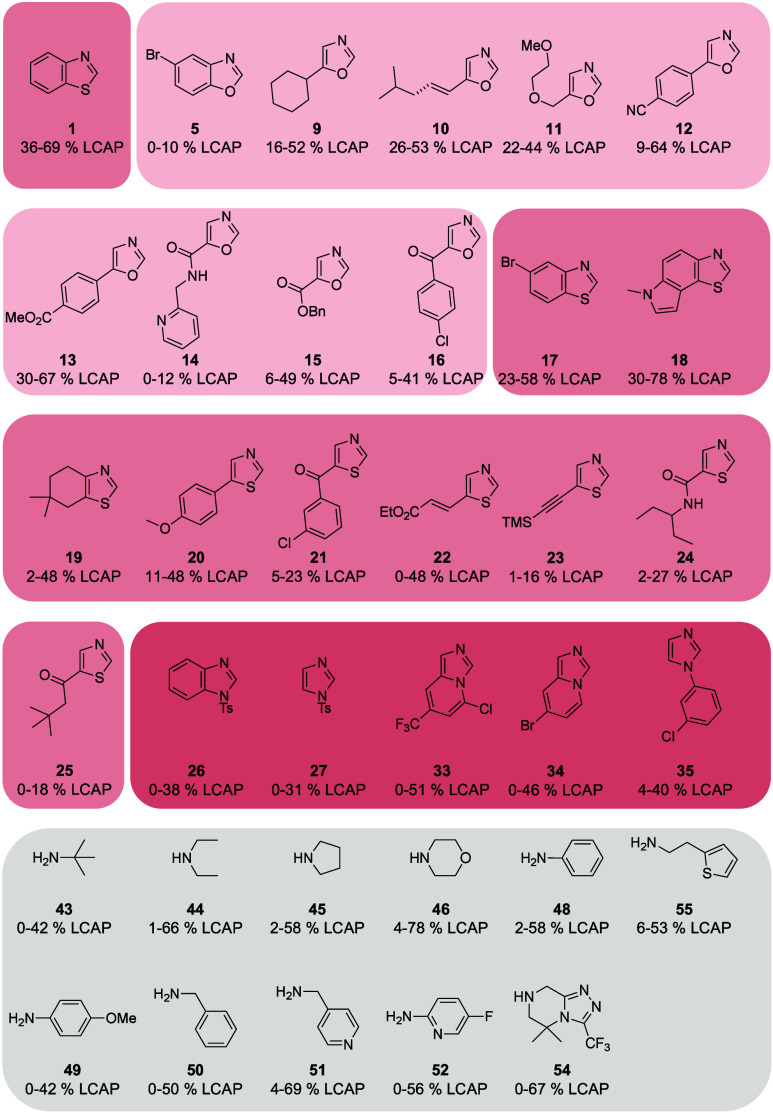
1,3-azoles (red boxes) and amines (gray box) considered in this work. The numbering was adopted from Felten et al.^19^ Oxazoles are displayed in light red, thiazoles in medium red, and imidazoles in dark red.

By utilizing computer-aided synthesis planning (CASP) tools, time and resources can be saved by focusing on the most promising synthesis routes or adapting protocols to achieve the desired outcome.^20−28^ Building on the data set of Felten et al. (Figure 3) and its associated synthesis protocol, we aim to develop an efficient CASP tool to assist synthetic chemists in expanding the chemical space of C2-carboxylated 1,3-azoles by predicting reaction yields (Figure 2).

Machine learning offers a suitable framework for developing such a CASP tool, assuming sufficient data is available.^20,29−31^ To illustrate the current state of CASP tools incorporating machine learning for yield prediction, examples from the literature are summarized in Table 1. However, the models referred to in this table are often not readily interpretable due to the complexity of their underlying architectures.^32^

**Table 1:** Overview of Previous Machine-Learning-Based Yield Prediction Studies

publication	model architecture	data set	# reactions	RMSE	*R*^2^	ref.
Ahneman et al.	Random Forest	Buchwald–Hartwig (BH) Reactions	4 608	7.8%	0.92	(33)
Granda et al.	Neural Network	Suzuki–Miyaura Reactions	5 760	11%		(34, 35)
Nielsen et al.	Random Forest	Deoxyfluorination	640	7.4%	0.93	(36)
Jiang et al.	Neural Network	USPTO Patent	269 132	23.0%		(37)
Yarish et al.	Neural Network	Enamine Frequent Reactions	29 904		0.18	(38)
Saebi et al.	Random Forest	BH Reactions from AstraZeneca	781		0.27	(39)
Chen et al.	Multimodal Model	Amide Coupling Reactions	41 239	19.3%	0.26	(40)
Schleinitz et al.	Random Forest	Nickel-Catalyzed Cross-Couplings	1 406	22.6%	0.54 (*r*^2^)	(41)

Interpretable models have the advantage that prediction outcomes can be directly linked to the input data,^42^ enabling human experts to draw conclusions or develop hypotheses that rigid models designed for specific tasks cannot provide. This linkage can be achieved using models whose coefficients quantify the influence of specific chemical features or properties on the prediction outcome in combination with explainable descriptors.^32,43^ The simplest and most interpretable class of models comprises linear models, which can be further differentiated by the objective function used to determine their optimal coefficients. Examples include least-squares, regularized least-squares (also known as “ridge”) and the least absolute shrinkage and selection operator (LASSO).^44^

These models contrast with nonlinear “black-box” models where the prediction outcomes cannot as easily be linked to the influence of the input data but often achieve better performance on more complex data sets.^32^ Examples of such model architectures include neural networks and Gaussian processes.^32^ The latter, however, offer partial interpretability by providing predictive probability distributions instead of single-valued predictions.^32,45^ The width of each distribution serves as a measure of the model’s uncertainty about its own predictions.

One way to determine the importance of features and influence of such “black-box” models is the SHapley Additive exPlanations (SHAP) method.^46^ This method requires a specified model and assigns each feature a value that quantifies its importance. SHAP values are determined by training a series of models, where one feature is excluded in each iteration. The prediction outcomes of these models are then compared to the prediction outcome of the original model, where all features are included.

In this work, we develop a yield prediction model based on the data set from Felten et al.^19^ to support synthetic chemists in expanding the chemical space of C2-carboxylated 1,3-azoles. To ensure interpretability, we integrate a heat-mapping algorithm named PIXIE (Predictive Insights and Xplainability for Informed chemical space Exploration), which visualizes the relationship between structural motifs and prediction outcomes. We apply our CASP tool featuring PIXIE to the ZINC database, demonstrating its practical utility in molecular design and targeted expansion of the chemical space of C2-carboxylated 1,3-azoles and beyond.

## 2 Methods

### 2.1 Data

#### 2.1.1 Labeled Data from Merck & Co

In the synthesis optimization study by Felten et al., LCAP yields of the coupling of 24 azoles with 12 amines were reported.^19^ The synthesis of amine **47** followed a different protocol than the standard one used for the other reagents, leading to the exclusion of its yields from our analysis. This results in 264 (instead of 288) data points for our study. The Cartesian nuclear coordinates and SMILES^47^ codes of the reactants were created manually based on the data given in the publication. The Cartesian nuclear coordinates were additionally subjected to a force field optimization.

#### 2.1.2 Unlabeled Data from ZINC20

The ZINC20 “In-stock” database was downloaded at 2023/11/02 and preprocessed according to our previous work.^15^ This database contains molecules listed as “purchasable” from different vendors. Based on this preprocessed database, a substructure search was performed to identify 1,3-azole amides. SMARTS codes (O=C(N)c1nccs1, O=C(n)c1nccs1, O=C(N)c1ncco1, O=C(n)c1ncco1, O=C(N)c1nccn1, and O=C(n)c1ncn1) were used for this purpose. The resulting subset contains 5708 1,3-azole amides on which predictions can be performed.

### 2.2 Descriptors

We employed different types of descriptors (Table 2) to determine the most suitable ones for yield prediction and interpretation. In this way, we are covering a wide range of descriptors with different advantages and disadvantages. A full list of the descriptors employed in this work can be found in the Supporting Information (see results-of-grid-search.xlsx, which is available at https://git.rz.tu-bs.de/proppe-group/yield-prediction).

**Table 2:** Overview of Descriptors Used in This Work

descriptor type	# descriptors	cost
RDKit Descriptors	209	+
QM Descriptors	5	++++
Fingerprints	3	++
Many-Body Descriptors	3	+++

#### 2.2.1 Property Descriptors

##### 2.2.1.1 From RDKit

All RDKit descriptors were calculated from the SMILES^47^ strings of the amide-coupling products using the RDKit Python toolkit (version 2023.03.1b1).^48^ The rdkit.Chem.Descriptors module from RDKit contains 209 molecular properties, which are widely used as features in chemical machine learning.^49^ These descriptors capture properties such as molecular weight, counts of different functional groups, partial charges, and relative atomic masses.^48^ Due to their manageable complexity, we primarily used RDKit molecular property descriptors to represent the chemical space in this project employing principal component analysis (PCA) for dimensionality reduction.

##### 2.2.1.2 QM-Derived

Quantum mechanics (QM)-derived descriptors provide detailed information on the electronic properties of molecules and the energetics of chemical reactions, making them valuable for machine learning. While generating QM descriptors is computationally expensive and limits the model’s ability to make fast (ideally “real-time”) predictions, their accuracy and detail can outweigh this drawback.

To include information about the reaction mechanism, the Gibbs free energy (Δ*G*) of the product formation step was calculated and used as a descriptor. This step was chosen because it directly involves the amine, ensuring that each product in the data set has a unique Δ*G* value. In contrast, Δ*G* values for other steps in the mechanism are indistinguishable across products derived from the same azole and different amines. Additional QM-derived descriptors include electronic properties such as HOMO and LUMO energies, dipole moments, electronegativity, and hardness of the reaction products.

QM-optimized structures and properties were obtained from a series of calculations, the first step of which was a conformational search using CREST^50^ (version 2.12) with the GFN2-xTB method.^51^ The lowest-energy conformer was used as input for subsequent density functional theory calculations performed in ORCA 5.0.^52,53^ We employed the B3LYP^54^ functional incorporating D3(BJ)-type^55,56^ dispersion corrections, balancing computational efficiency with structural and energetic accuracy.^57^ Initial optimizations used the def2-SVP basis set,^58^ followed by refined optimizations with def2-TZVP.^58,59^ Harmonic frequency calculations confirmed structural minima and provided Δ*G* values (cf. Equation 7 in ref. (60)). Structures with imaginary frequencies below 100 cm^–1^ were considered optimized, as such frequencies are treated as artifacts.^59^

To account for solvation effects, the CPCM solvation model was employed.^61^ The solvent mixture used in the product-forming step, as described by Felten et al., consists of 1,2-dimethoxyethane (DME) and ethyl acetate (EtOAc) in a 1:4 ratio.^19^ The dielectric constant (6.26) and refractive index (1.374) of this mixture were calculated as weighted averages of the pure solvents’ dielectric constants (DME, 7.20; EtOAc, 6.02) and refractive indices (DME, 1.380; EtOAc, 1.372).^62−65^ The temperature was set to 303.15 K, consistent with the experimental conditions of the amide coupling step.^19^

#### 2.2.2 Structural Descriptors

##### 2.2.2.1 Fingerprints

Molecular fingerprints are one way to encode molecules as binary vectors that represent their chemical structure. Following the approach by Haywood et al.,^66^ three different types of fingerprints were used for each amide-coupling product: the MACCS fingerprint,^67^ the RDK fingerprint,^48^ and the Morgan fingerprint.^68^

The fingerprints were calculated from the SMILES codes of the amide-coupling products using the RDKit Python toolkit (version 2023.03.1b1).^48^ A fingerprint length of 2048 bits was used for the RDK and Morgan fingerprints, with a radius of 2 for the Morgan fingerprint. All other settings were kept at their default values.

The RDK fingerprint is based on bond paths and, in this case, includes substructures containing up to seven bonds.^48^ These substructures are transformed into “on bits” within the fingerprint using a hash function. While the use of a hash function prevents the direct reconstruction of a molecule from its fingerprint, information about which substructure corresponds to which bit can be retrieved through the bitInfo map during fingerprint generation.

##### 2.2.2.2 Many-Body Descriptors

Many-body descriptors, which encode and differentiate between the three-dimensional structural features of a molecule, provide more steric information than property descriptors or fingerprints. However, these descriptors are generally less interpretable.

The many-body descriptors considered in this work were calculated on the QM-optimized structures of the reaction products resulting from the protocol described in Section 2.2.1. While this may increase the accuracy in comparison to learning on unoptimized structures, this step also increases the computational cost of the descriptor generation process. In cases where optimized structures are not already available as a byproduct of, e.g., QM-derived property descriptors, it is advisible to examine the suitability of unoptimized structures for generating many-body descriptors.

The Coulomb Matrix (CM) is constructed from atomic energies (diagonal elements) and the internuclear Coulomb potential, which depends on the charges of and distances between atomic nuclei (off-diagonal elements).^69^ Here, we considered the sorted eigenvalues (by absolute value) of the CM.

The “two-body forces” descriptor *F*_2B_ by Pronobis et al. is also built upon the Coulomb potential but distinguishes between unique pairs of chemical elements.^70^ For instance, all inverse hydrogen–carbon distances are summed into the same feature of the *F*_2B_ vector. In the original implementation, each unique element pair was assigned 15 features, corresponding to different orders of the inverse distance (from 1 to 15) to increase the descriptor’s flexibility. Here, we reduced computational cost by introducing only one feature per unique element pair, using an order of 1 as in the Coulomb potential.

The many-body tensor representations (MBTR) descriptor provides a discretized spectrum of one-body (atomic numbers), two-body (distances), and three-body (angles) features.^71^ Each feature type occupies a separate segment of the spectrum. Atomic numbers, distances, and angles are first smoothed using Gaussian functions and then discretized into a finite vector. In this work, the MBRT descriptor was used with default settings, except for the number of discrete grid points, which was set to 20.

### 2.3 Model Building

The Python library scikit-learn (Version 1.3.2) was used for all model-building steps described in this work.^72^ To predict the LCAP yields, various regression models were evaluated: Gaussian process regression (GaussianProcessRegressor()) with the kernel ConstantKernel() * Matern()+WhiteKernel(), five restarts of the optimizer (n_restarts_optimizer=5), and normalized target values (normalize_y=True), gradient boosting regression (GradientBoostingRegressor()), linear least-squares regression (LinearRegression()), Bayesian ridge regression (BayesianRidge()), LASSO regression (Lasso()), multilayer perceptron regression (MLPRegressor()), and random forest regression (RandomForestRegressor()). These methods were selected for their balance between predictive power, computational efficiency, and widespread use in the field.^73,74^

To find the best model architecture and descriptor combination, a grid search was performed using all descriptors listed in Section 2.2 and Table 2. The grid search results are provided in the Supporting Information (SI-Grid-Search.xlsx).

#### 2.3.1 Data Set Preparation and Splitting

The data set preparation was standardized to ensure consistency across models. A test set containing 50 samples (20% of the data) was defined, leaving the remaining 214 data points for training and validation. For validation, the leave-one-out (LOO) method was used, resulting in 214 separate models, each trained on all but one data point. This method allowed the identification of the best-performing model and descriptor combination. Additional details on the data splitting strategy can be found in Section S2. Negatively predicted yields were set to 0 in all cases.

#### 2.3.2 Model Performance Evaluation

Model performance was evaluated using several metrics, including the median (AE_50_, eq 1), the mean absolute error (MAE, eq 2), the root-mean-square error (RMSE, eq 3), the maximum absolute error (A*E*_max_, eq 4), and the coefficient of determination (*R*^2^, eq 5).
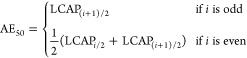
1
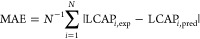
2
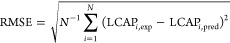
3
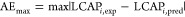
4
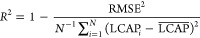
5

## 3 Results and Discussion

### 3.1 Yield Prediction

Table 3 lists the best performing descriptor for each regression type as measured by the MAE resulting from a LOO analysis of the training set. Multilayer perceptron regression plus Morgan fingerprint and Bayesian ridge regression plus RDK fingerprint perform identical according to this statistic, with a MAE of 7.1 yield percent. Due to the linearity of its predictive function, the Bayesian ridge model has the advantage of being more interpretable than the multilayer perceptron, which is a feedforward neural network with nonlinear activation functions. The following analysis pertains to the Bayesian ridge–RDK fingerprint model unless stated otherwise.

**Table 3:** Performance of the Best Descriptors for Each Regression Model Based on the MAE in 214 LOO Models

regression type	best descriptor	AE_50_ [%]	MAE [%]
Linear Least Squares	MACCS Fingerprint	8.1	9.6
LASSO	RDK Fingerprint	5.9	7.9
Bayesian Ridge	RDK Fingerprint	5.6	**7.1**
Random Forest	RDK Fingerprint	5.4	7.3
Gaussian Process	MACCS Fingerprint	7.4	8.5
Gradient Boosting	Morgan Fingerprint	5.5	7.2
Multi-Layer Perceptron	Morgan Fingerprint	5.6	**7.1**

**Figure 4:**
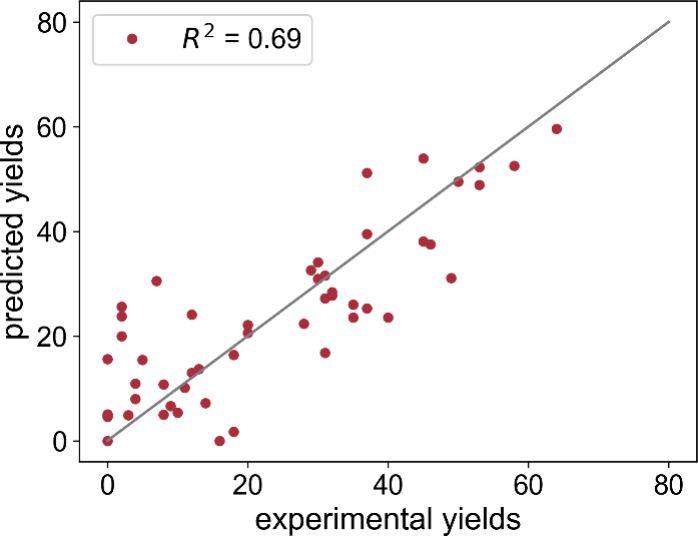
Agreement between the predicted and experimental LCAP yields of the test set. The gray line represents ideal agreement, where predictions perfectly match the experimental values.

The MAE on the test set equals 7.4 yield percent (Table 4). The largest prediction errors occur at the lower end of the yield scale, whereas more accurate predictions are made at the upper end of the scale (Figure 4), which we consider a fortunate trend. The maximum absolute error (A*E*_max_), an estimate of the worst-case scenario, amounts to 24 yield percent. While this error might be considered substantial, the model still appears to reliably predict the general direction of the reaction outcome.

**Table 4:** Performance of the Test Set (50 Molecules) and Fivefold Cross-Validation on the Training Set (214 molecules) as Described in Section 2.3

	*R*^2^	MAE	RMSE	AE_max_
test set	0.69	7.4%	9.9%	23.6%
first fold	0.67	7.7%	10.0%	26.8%
second fold	0.72	5.8%	8.0%	23.6%
third fold	0.80	5.1%	6.8%	21.9%
fourth fold	0.63	7.0%	9.3%	30.8%
fifth fold	0.66	8.1%	10.7%	27.8%
mean folds	0.70	6.7%	8.9%	26.2%
std folds	0.06	1.1%	1.4%	3.14%

To ensure that the model is not biased toward the test set, a 5-fold cross-validation was performed on the training set (Table 4). The results, with an average MAE of 6.7% and a standard deviation of 1.1%, suggest that this specific type of bias is negligible.

Unlike the LCAP yields analyzed in this study, many synthetic laboratories commonly report isolated yields as a measure of reaction efficiency. To investigate the relationship between these two quantities, we examined the correlation between LCAP yields and isolated yields. In the study by Felten et al.,^19^ 24 1,3-azoles were coupled with amine **50**, and both LCAP and isolated yields were reported for these reactions. Notably, in all but one case, the isolated yields exceeded the LCAP yields (Figure S4). This observation suggests that the yields predicted by our model, based on LCAP data, are likely to reflect a conservative estimate, with isolated yields potentially being equal to or higher than the predicted values.

### 3.2 Strengths and Limitations of Our Model

Previous data sets used for training yield prediction models (Table 1) are generally more comprehensive than the training set of 214 reactions by Felten et al.^19^ (Merck & Co.) examined in this work. While the performance metrics of our model surpass those reported for the models developed by Granda et al.,^34,35^ Jiang et al.,^37^ Yarish et al.,^38^ Saebi et al.,^39^ Chen et al.^40^ and Schleinitz et al.,^41^ it is important to note that these models were evaluated on broader data sets covering a more diverse chemical space. The higher metrics observed in this study are likely due to the system-focused nature of the Merck data set (Figure 3), which narrows the scope of predictions. This specificity improves accuracy within the data set but comes at the cost of generalizability, as our model is not expected to perform well on reactions outside this limited chemical space.

Ahneman et al. reported performance metrics for a similarly small training set of 230 Buchwald–Hartwig couplings.^33^ Their model achieved an *R*^2^ of 0.68 and an RMSE of 15.3%, which is comparable to the performance of our model (*R*^2^ = 0.69, RMSE = 9.9%, cf. Table 4). The performance gap relative to their full model (*R*^2^ = 0.92, RMSE = 7.8%, see first entry in Table 1) highlights the potential for improved accuracy in our model if additional data were available for training.

While efforts were made to ensure that the test set is representative of the training set (Figure S2, right), the combinatorial nature of the Merck & Co. data set poses an additional challenge, as it inevitably results in shared structural motifs between the training and test sets. This overlap limits the model’s ability to predict yields for entirely new combinations of azoles and amines (Figure 3). To quantify this limitation, out-of-sample predictions were conducted in a leave-one-compound-out (LOCO) fashion. In each iteration, the model was trained on all but one azole and subsequently used to predict the yields for the excluded azole. The same procedure was applied to the amines. Table 5 summarizes the five best (0.68 ≤ *R*^2^ ≤ 0.91) and five worst (−6.8 ≥ *R*^2^ ≥ – 51.0) LOCO performances. The complete table can be reproduced using the code provided in the SI.^75^

**Table 5:** Performance of LOCO Models on Unseen Azoles and Amines

structure	*R*^2^	MAE	RMSE	AE_max_
**50**	0.91	3.1%	4.0%	9.1%
**48**	0.73	7.4%	9.1%	23.6%
**49**	0.72	5.1%	7.0%	18.1%
**51**	0.70	7.0%	9.8%	22.9%
**44**	0.68	7.0%	10.2%	26.2%
**22**	–6.8	14.4%	15.4%	19.7%
**25**	–6.9	12.8%	12.8%	15.0%
**23**	–15.4	14.8%	16.4%	26.2%
**5**	–45.9	22.0%	22.5%	33.7%
**14**	–51.0	25.1%	26.0%	37.6%

Among the top-performing unseen structures, only amines are present. These amines include both aliphatic (**44**) and aromatic (**48**–**51**) compounds. This result suggests that the model is capable of making accurate out-of-sample predictions for unseen amines. In contrast, the predictive performance is notably low for certain azoles (**5**, **14**, **22**, **23**, **25**). In these cases, the model underperforms compared to a simple average of experimental yields.

The poor predictions for **22**, **23**, and **25** can be attributed to how the molecules are encoded in the model. Since the RDK fingerprint is a substructure-based descriptor, it performs well on molecules composed of substructures already known to the model. However, this approach leads to errors when predicting yields for molecules with structural motifs unknown to the model. For instance, **23** contains a trimethylsilane (TMS) group, a motif absent from the training data set in the LOCO training cycle. As a result, the model is unable to make accurate predictions for such molecules.

**Figure 5:**
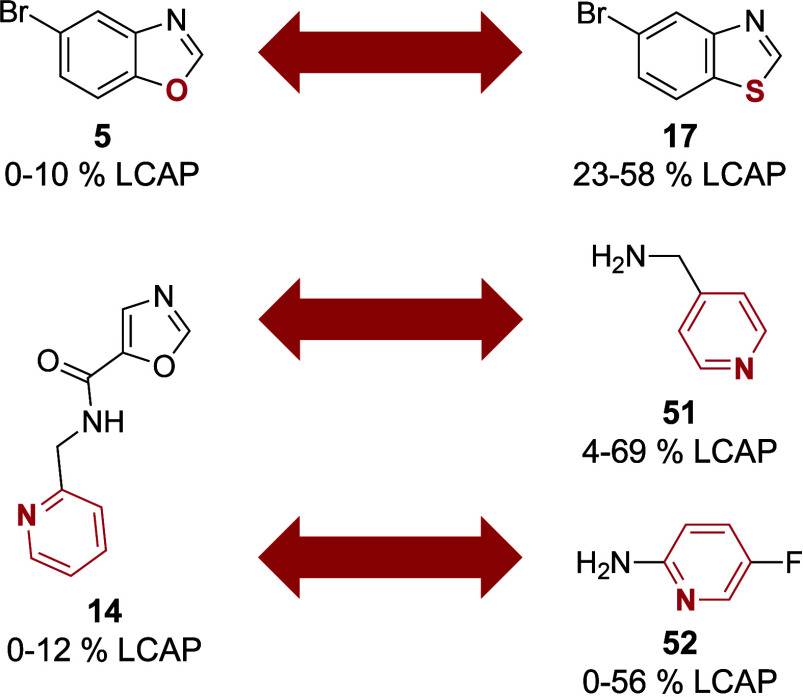
Structures with similar structural motifs but distinctly different yields. The structure motifs of interest are highlighted in red.

For **5** and **14**, similar structures in the training data set exhibit distinct yields. For example, **5** (an oxazole) and **17** (a thiazole) differ only in the heteroatom of the azole motif (Figure 5, top). However, **17** achieves yields ranging from 23% to 58%, whereas **5** produces a maximum yield of only 10%. A similar pattern is observed for **14**, which contains a pyridine motif. This motif is also found in amines **51** and **52**, yet their reaction products yield up to 69%, while products containing **14** achieve a maximum yield of only 12% (Figure 5, middle and bottom).

### 3.3 Learning from Our Model

The discrepancies observed for azoles **5** and **14** are reminiscent of the concept of *activity cliffs*, a phenomenon commonly discussed in medicinal chemistry and drug design. Activity cliffs describe structurally similar compounds that exhibit different biological activities.^76^ This concept can be generalized to other properties, such as reaction yields, where such differences are referred to as property cliffs.^77^

Property cliffs can be investigated through an analysis of the model’s bits {*x*_*n*_} and coefficients {ω_*n*_},
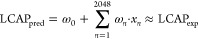
6

Each bit is linked to a model coefficient. The coefficients indicate the contribution of each substructure to the predicted yield given they are linked to “on” bits (*x*_*n*_ = 1), as opposed to “off” bits (*x*_*n*_ = 0). Positive coefficients signify a positive influence on the yield. Negative coefficients denote a negative influence. The magnitude of the coefficient reflects the overall impact of the substructure. The individual coefficients of our model can be printed out with the code provided in the project-related Gitlab repository.^75^

To further understand the discrepancies observed for azoles **5** and **14**, we compared the coefficients from models trained on LOCO subsets to those from a model trained on the entire data set. The analysis focused on “on” bit coefficients with the largest differences between the two models. For instance, the oxazole motif in **5**, which distinguishes it from **17** containing the analogous thiazole motif (Figure 5, top), was associated with the most significant coefficient changes. This finding suggests that such changes can result from the descriptor’s inability to consistently capture the influence of certain motifs, even though similar structures containing the same motifs are present in the training set.

The analysis of coefficient–bit pairs is not only useful for understanding the aforementioned discrepancies. It also serves as a powerful tool for addressing broader interpretability challenges in machine-learning models. By linking specific structural motifs to their contributions, this approach provides valuable insights into the underlying patterns driving predictions.

To make the information provided by the model more accessible and intuitive, we implemented the heat-mapping algorithm PIXIE to visualize the importance of individual features as measured by their associated coefficients (eq 6). Using this information, we assigned weights to each bond in a molecule by summing the coefficients of all substructures associated with that bond. These weights were then visualized as colors on the molecular structure, creating a heat map. In the representation used here, red tones indicate a positive influence of the structural motif on the yield, while blue tones suggest a negative influence. The color scale was normalized to the largest and smallest weights in the data set for consistency.

**Figure 6:**
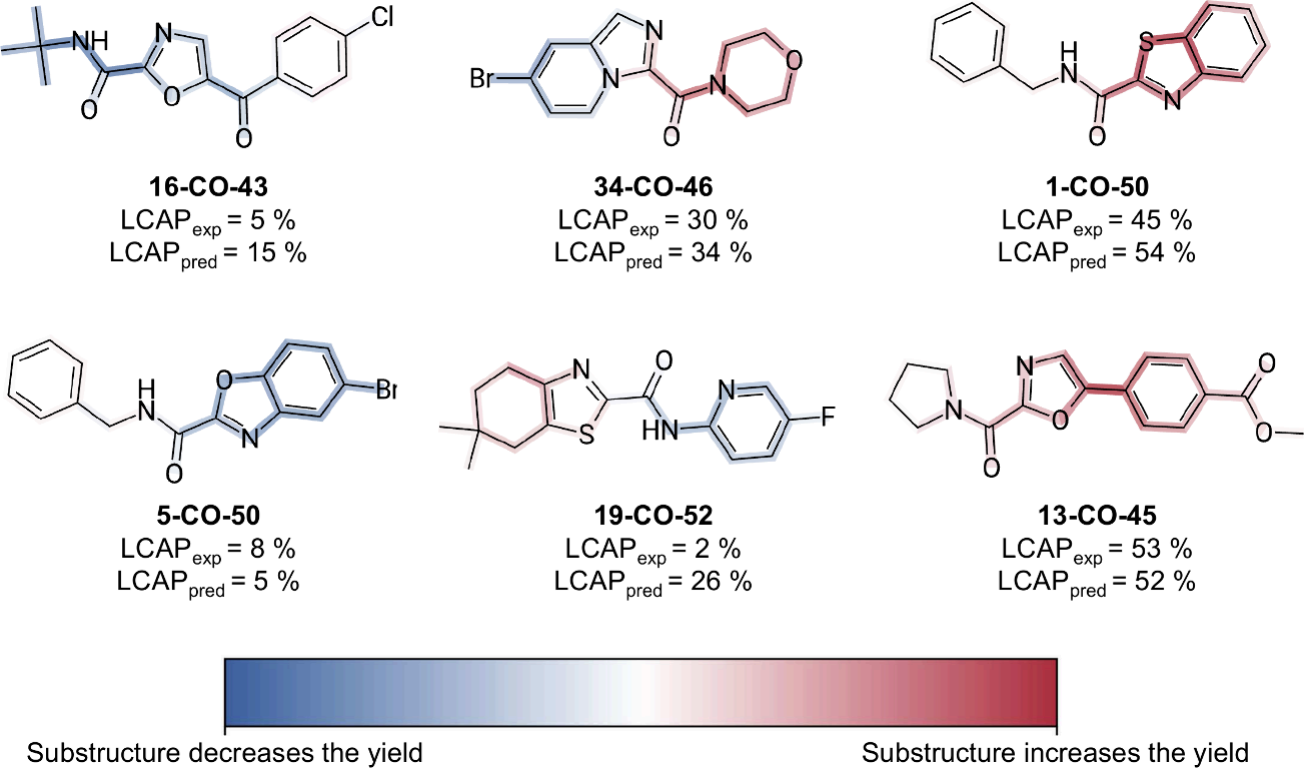
Heat maps showing examples from the test set. Blue tones indicate substructures that reduce the predicted yield, while red tones highlight those that increase it.

For example, as shown in Figure 6, the brominated oxazole substructure in **5-CO-50** and **34-CO-46** is identified as detrimental to the yield. Conversely, the 5-phenyl oxazole substructure in **13-CO-45** is linked to a positive strong contribution. The analysis also highlights the role of specific amines. For instance, amine **46** shows a potential positive effect on the yield of **34-CO-46**, while amine **50** appears to contribute negatively to the yield of **1-CO-50**.

While the heat maps generated by PIXIE offer valuable insights, inconsistencies may arise due to fingerprint collisions—instances where a single bit corresponds to multiple substructures. Such collisions can hinder the unambiguous assignment of coefficients to specific substructures within a molecule, limiting the interpretability of the heat map.^78^ Due to the narrow scope of the chemical space investigated in this work, such collisions are expected to have a negligible impact. Nevertheless, we note this limitation to ensure the approach remains adaptable and informative in broader applications beyond the specific system studied here.

Heat-mapping algorithms have already been used in similar contexts to visualize how machine-learning models make predictions.^78−83^ These methods fall under the broader domain of explainable artificial intelligence and are often applied in drug design.^78−82^ For instance, Riniker et al. implemented a heat-mapping approach in RDKit, focusing on mapping based on molecular similarity or the predicted probability of a machine-learning model trained on fingerprints.^78^ Marcou et al. developed an approach named ColorAtom for explainability based on fragment descriptors.^84^ PIXIE, on the other hand, is based on RDKit fingerprints and can therefore be applied to other types of models.

PIXIE has the distinct advantage of being directly applicable to model coefficients, which is not feasible with more complex model architectures. For such architectures, tools like SHAP^46^ are often used to explain predictions. PIXIE was designed to include SHAP values for models trained on RDKit fingerprints, enabling the generation of comparable heat maps (Figure S5).

### 3.4 Exploring the 1,3-Azole Amide Space

**Figure 7:**
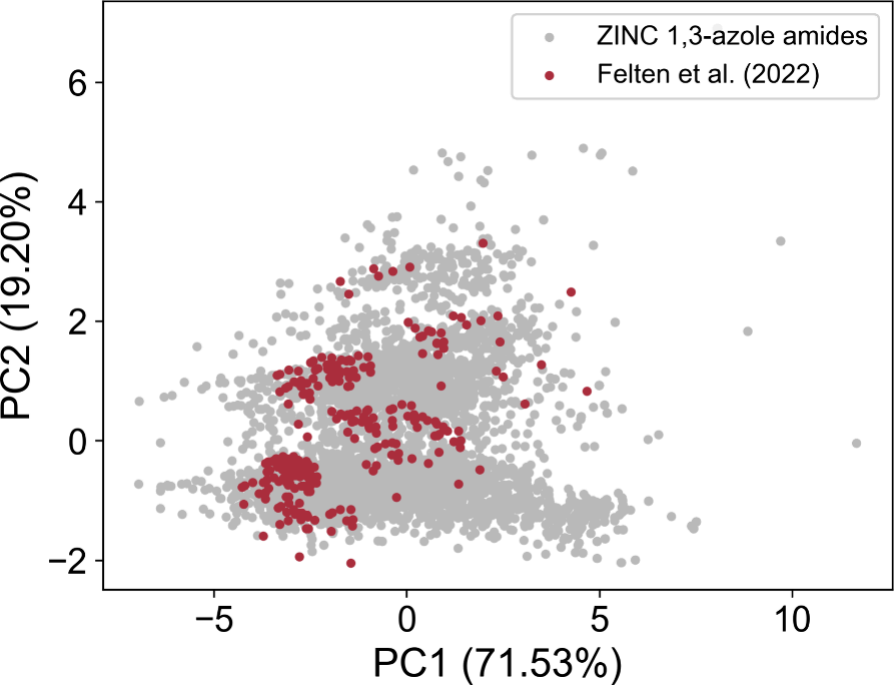
PCA based on seven RDKit descriptors, with the data by Felten et al.^19^ shown in red and the 1,3-azole amides from the ZINC database in gray. Details on the PCA settings are provided in Section S1.

To explore the applicability of our machine-learning model to “real-world” data, we conducted yield predictions for azole amides from the ZINC20 database (Section 2.1.2). This database includes 5708 1,3-azole amides in its “In-Stock” subset, with predicted yields ranging from 9% to 60%. The Merck & Co. data set (Section 2.1.1) used to train the model provides only partial coverage of the structural diversity found in the ZINC database, as illustrated in Figure 7. As a result, predictions for structures located further from the training set in the chemical space, depending on the chosen descriptor, may be less accurate than those closer to it.

This trend is also reflected in the prediction uncertainty, which generally increases for structures farther from the training set. However, in this case, prediction uncertainties span a relatively narrow range of 8% to 10%, suggesting consistent model confidence across the data set. Examples of ZINC structures, along with their predicted yields and associated uncertainties, are summarized in Table 6.

**Table 6:**
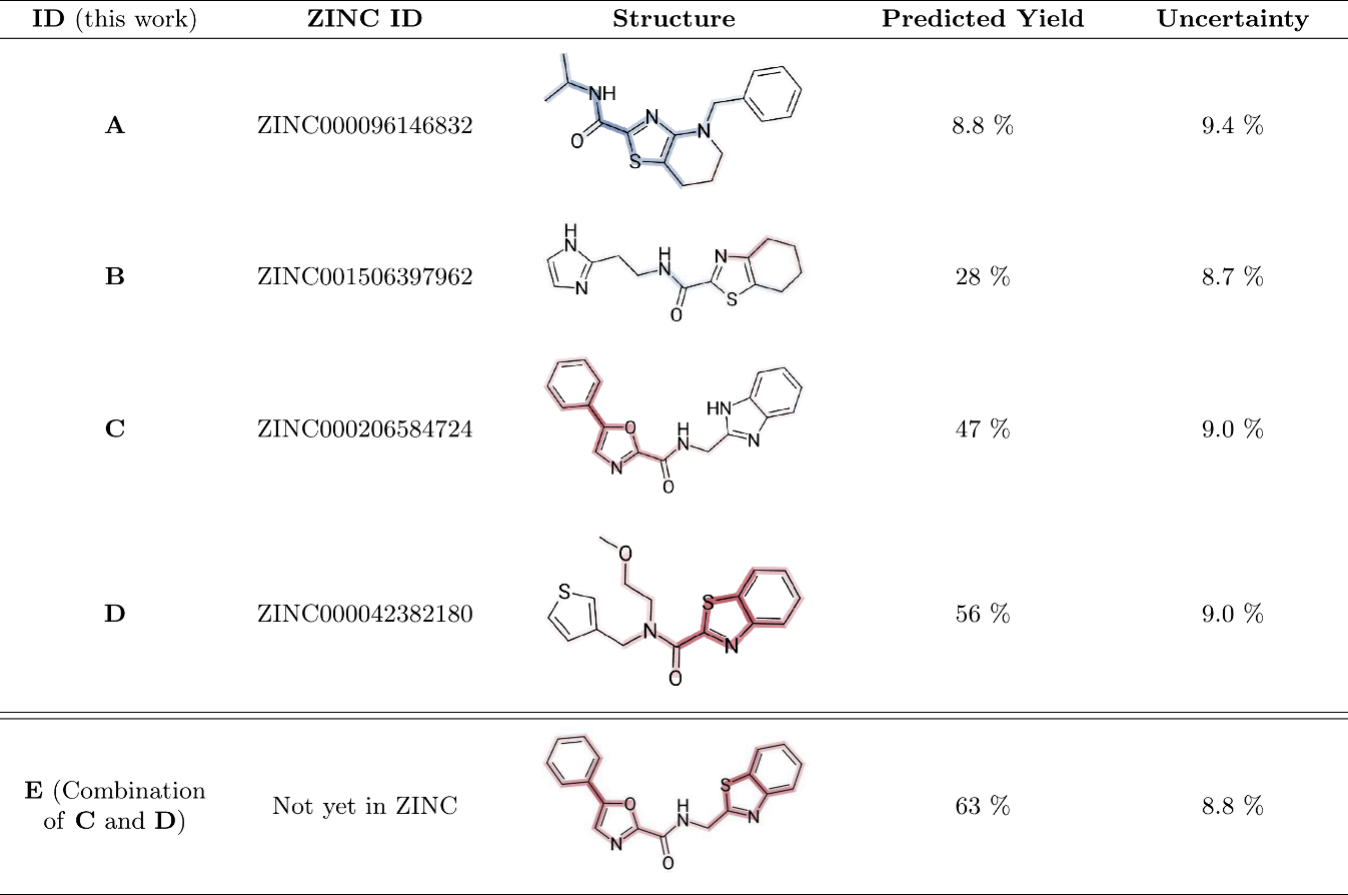
Predicted Yields and Uncertainties for Selected Molecules from the ZINC20 Database and a Newly Designed Molecule (**E**)^a^

a Blue regions on the structures indicate substructures predicted to decrease the yield, while red regions highlight those predicted to increase it. Molecule **E**, designed by combining motifs from **C** and **D**, is not part of the ZINC database. Uncertainty values for each prediction are listed in the corresponding column.

The predictions in Table 6 suggest that structure **C**, which includes a 5-phenyl-1,3-oxazole group, is associated with a positive impact on the yield, while the benzoimidazole group appears to have a neutral effect. In contrast, structure **D** contains a benzothiazole group that is predicted to enhance the yield. By replacing the benzoimidazole group in structure **C** with the benzothiazole group from structure **D**, a new molecule—structure **E**—was designed, combining two motifs predicted to positively influence the yield. It is important to note, however, that this analysis is purely mathematical and does not account for chemical feasibility. In practice, the substitution of these motifs may not be straightforward, and alternative reaction mechanisms could affect the validity of the model’s predictions. Thus, the conclusions drawn here are only valid under the known assumptions of the model. This example nonetheless demonstrates how heat mapping can support the rational design of molecules.

## 4 Conclusions

The chemical space of C2-carboxylated 1,3-azoles remains largely underexplored. To facilitate systematic exploration, we developed an interpretable machine-learning model for yield prediction using RDK fingerprints. The model targets C–H carboxylation reactions followed by an amide coupling step and achieves a test set MAE of 7.4 yield percent, which corresponds to an *R*^2^ value of 0.69.

Limitations such as property cliffs (analogous to activity cliffs) and underrepresented structural motifs were identified, highlighting areas for further refinement. To provide deeper insights into the model’s predictions, we integrated a heat-mapping algorithm named PIXIE. This algorithm enhances chemical intuition by visualizing the quantitative effect of individual structural motifs on the predicted yields, making the model predictions directly interpretable.

The adaptability of PIXIE extends beyond yield prediction, with potential applications in quantitative structure–property relationships (QSPR) for properties like quantum chemical descriptors and in quantitative structure–activity relationships (QSAR) for activities or toxicities. By demonstrating its utility on real-world data from the ZINC database, we showed how PIXIE can facilitate rational molecular design.

By combining predictive modeling with interpretable visualizations through PIXIE, we aim to support synthetic chemists in systematically expanding the chemical space of C2-carboxylated 1,3-azoles and beyond.

## Data Availability

The data used for creating the machine-learning model can be found under https://pubs.acs.org/doi/abs/10.1021/jacs.2c10557. The ZINC “in-stock” can be downloaded via https://zinc.docking.org/tranches/home/. The code as well as the model to reproduce the results shown in this work are available at https://git.rz.tu-bs.de/proppe-group/yield-prediction.

## Supporting Information Available

Additional information regarding the principal component analysis (PCA), the evaluation of the data set as well as additional figures on SHAP-based heat mapping, and the assessment of isolated yield (PDF)
Data of the grid search (XLSX)

## Abbrevations

AE_50_: Median of the Absolute Error
AE_max_: Maximum Absolute Error
BH: Buchwald–Hartwig
CASP: Computer-Aided Synthesis Planning
CM: Coulomb Matrix
DME: 1,2-Dimethoxyethane
EtOAc: Ethyl Acetate
HOMO: Highest Occupied Molecular Orbital
LASSO: Least Absolute Shrinkage and Selection Operator
LCAP: Liquid Chromatography Area Percent
LOCO: Leave-One-Compound-Out
LOO: Leave-One-Out
LUMO: Lowest Unoccupied Molecular Orbital
MAE: Mean Absolute Error
MBTR: Many-Body Tensor Representations
PCA: Principal Component Analysis
QM: Quantum Mechanics
*R*^2^: Coefficient of Determination
RMSE: Root Mean Square Error
SHAP: SHapley Additive exPlanations
TMS: Trimethylsilane
