# Supplementary material for: Predicting and Explaining Yields with Machine Learning for Carboxylated Azoles and Beyond
Source: J Chem Inf Model. 2025 Feb 7;65(4):1862–72. doi: 10.1021/acs.jcim.4c02336 (PMC11863374; doi:10.1021/acs.jcim.4c02336)
Supplement: Supplementary file 1 — ci4c02336_si_001.pdf [file ci4c02336_si_001.pdf]

Electronic Supporting Information for "Predicting and Explaining Yields with Machine Learning for Carboxylated Azoles and Beyond"

Kerrin Janssen and Jonny Proppe*

TU Braunschweig
Institute of Physical and Theoretical Chemistry
Gauss Str 17, 38106 Braunschweig, Germany

E-mail: j.proppe@tu-braunschweig.de

Additional supplementary information is available in our GitLab repository:
https://git.rz.tu-bs.de/proppe-group/yield-prediction

Section S1: Principal Component Analysis

To analyze the data and evaluate the different splits, a principal component analysis (PCA) was conducted. Prior to performing the PCA, a feature selection process was applied to the 209 RDKit descriptors, identifying a combination of descriptors with high explained variance. As a result, seven descriptors were selected for the PCA: Chi1v, Chi2n, Chi2v, Chi3v, Chi4v, PEOE_VSA5, and SMR_VSA5. The Chi descriptors represent various types of connectivity indices, PEOE refers to the Partial Equalization of Orbital Electronegativities, and the SMR descriptor is based on molecular refractivity.[S1-S3] VSA on the other hand stands for van der Waals surface area so the resulting descriptors PEOE_VSA5 and SMR_VSA5 are designed to describe the electrostatic interactions and polarizability of the molecule.[S4] The PCA was performed using the scikit-learn Python library (version 1.3.2).[S5] Before applying PCA, the selected descriptors were standardized using the "StandardScaler" function from scikit-learn.

Section S2: Evaluation of the Dataset

To estimate the validity of the chosen train–test split (80/20 ratio), a learning curve analysis was performed using Bayesian ridge regression on RDK fingerprints (Fig. S1). For this analysis, 100 models were trained on randomly selected data points for each training set size, and the median R^2 value (Eq. 5 in the main paper) of these models was evaluated on the fixed test set. The resulting learning curve (Fig. S1) confirmed that the chosen split provides sufficient data for robust model evaluation. For testing, 50 data points were randomly selected.

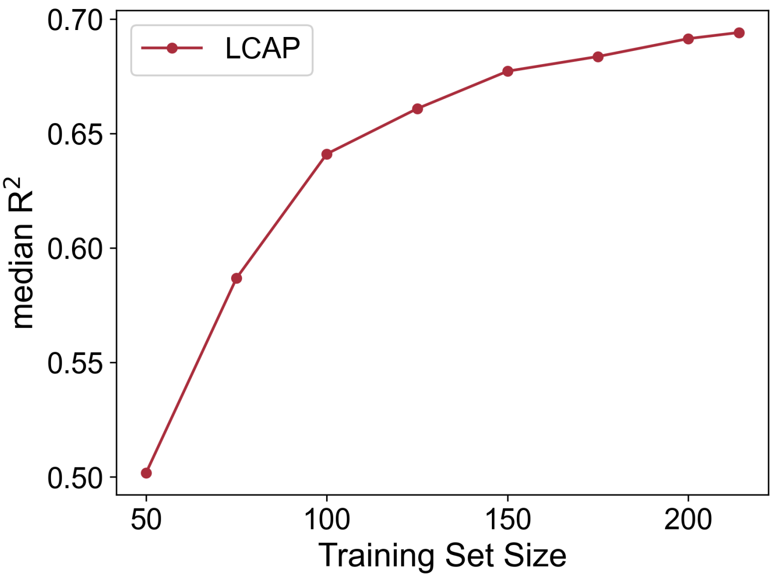

Figure S1: Learning curve of the model on LCAP yields using the RDK fingerprint as descriptor. The plot shows the median R^2 value of 100 randomly initialized Bayesian ridge regression models for each training set size.

To ensure a comprehensive representation of the chemical space, a PCA was conducted on both the training and test data. The data of the test set is well-distributed within the training set, highlighting the suitability of the selected test set (Fig. S2, left). The corresponding loading plot (Fig. S3) illustrates the influence of each descriptor. Additionally, the distribution of targeted LCAP yields across the training and test datasets was analyzed to ensure representativeness (Fig. S2, right). The analysis shows that each yield class is represented in the test set by at least one data point, except for the bin with the highest yields, which contains only a single data point. We therefore consider the train–test split appropriate for evaluating the model's predictive performance without introducing out-of-scope predictions in the test set.

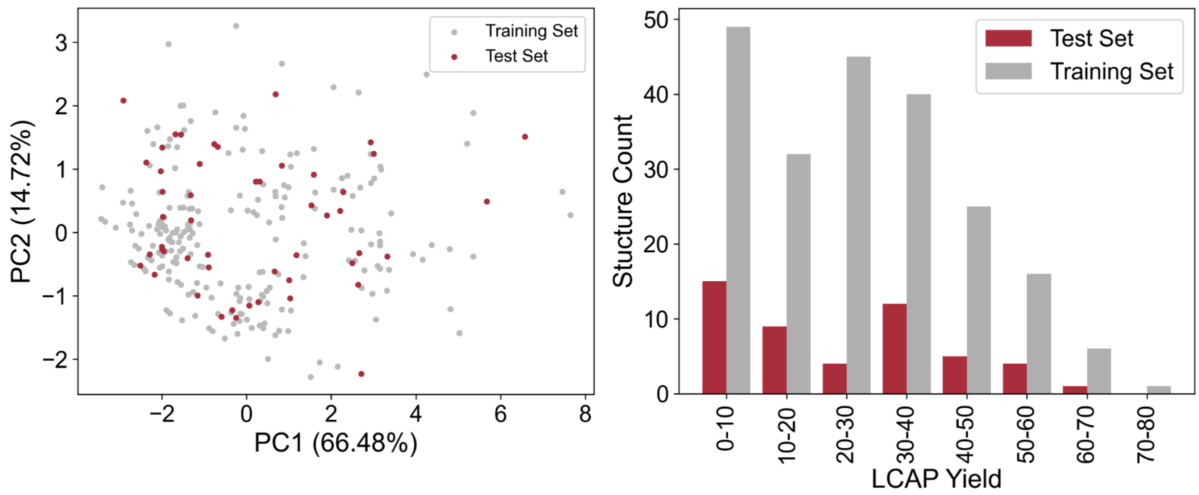

Figure S2: PCA based on seven RDKit descriptors (left) and histogram of the distribution of LCAP yields (right) in the test and training data. The 50 samples in the test set are shown in red, while the 214 structures in the training set are shown in gray.

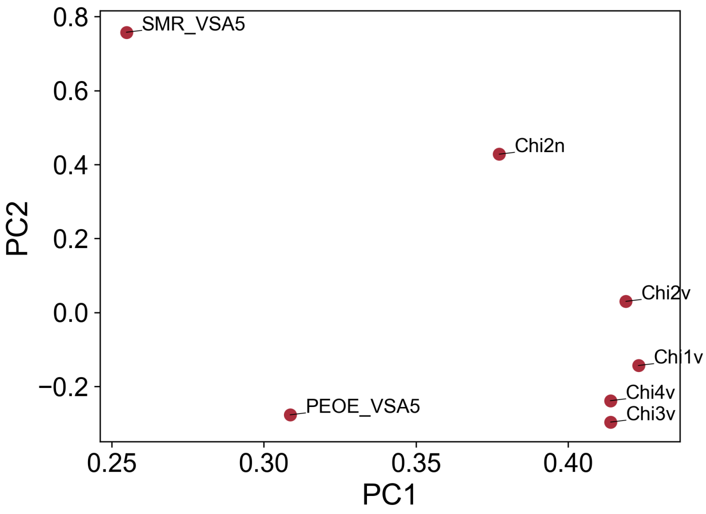

Figure S3: Loadings plot corresponding to the PCA shown in Figure S2 (left panel).

Section S3: Supporting Figures

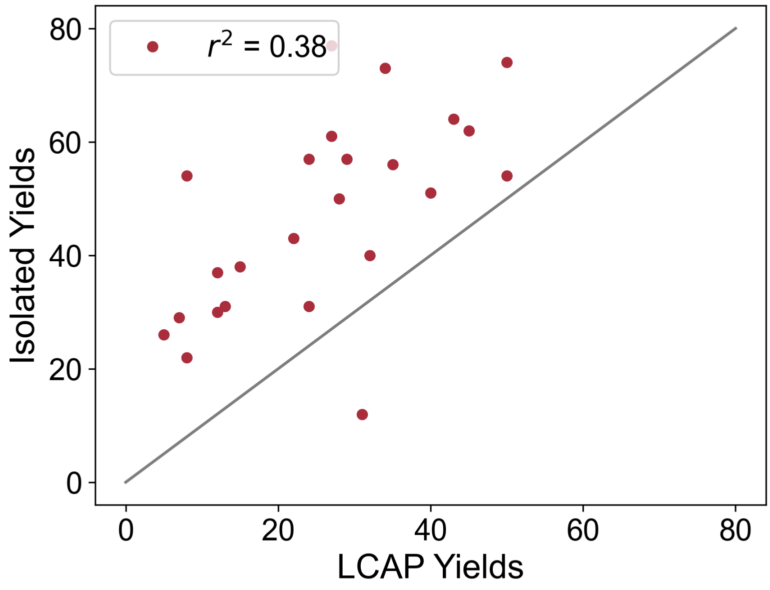

Figure S4: Correlation between isolated and LCAP yields of 24 1,3-azole amides from the work of Felten et al.[S7] as measured by Pearson's squared correlation coefficient. The gray line represents ideal agreement between both types of yield.

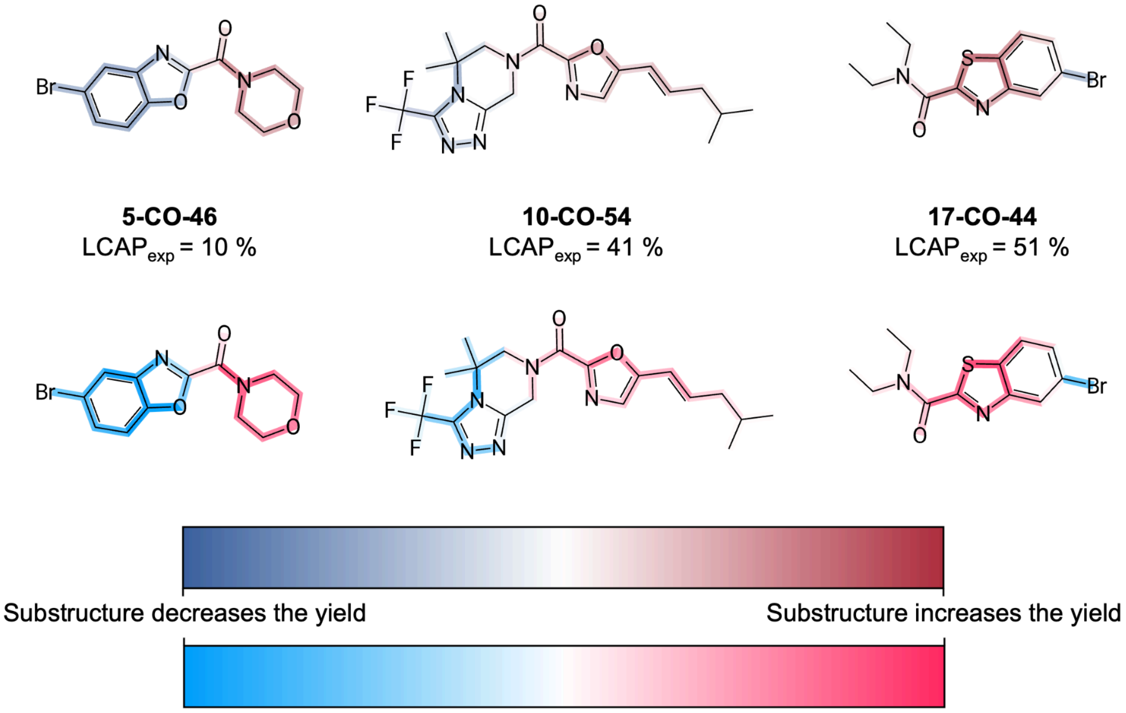

Figure S5: PIXIE-derived heat maps illustrating examples from the training set. The top row visualizes the model coefficients using a color scale from blue to red, while the bottom row shows heat maps based on SHAP values (blue to pink). SHAP values were computed using the linear explainer from SHAP version 0.44.1.

References

S1 L. H. Hall and L. B. Kier, “The Molecular Connectivity Chi Indexes and Kappa Shape Indexes in Structure-Property Modeling”, en, in Reviews in Computational Chemistry, Vol. 2, edited by K. B. Lipkowitz and D. B. Boyd, 1st ed. (Wiley, Jan. 1991), pp. 367–422.

S2 J. Gasteiger and M. Marsili, “Iterative partial equalization of orbital electronegativity—a rapid access to atomic charges”, en, Tetrahedron 36, 3219–3228 (1980).

S3 S. A. Wildman and G. M. Crippen, “Prediction of Physicochemical Parameters by Atomic Contributions”, en, Journal of Chemical Information and Computer Sciences 39, 868–873 (1999).

S4 P. Labute, “A widely applicable set of descriptors”, en, Journal of Molecular Graphics and Modelling 18, 464–477 (2000).

S5 F. Pedregosa, G. Varoquaux, A. Gramfort, V. Michel, B. Thirion, O. Grisel, M. Blondel, P. Prettenhofer, R. Weiss, V. Dubourg, J. Vanderplas, A. Passos, D. Cournapeau, M. Brucher, M. Perrot, and É. Duchesnay, “Scikit-learn: Machine Learning in Python”, Journal of Machine Learning Research 12, 2825–2830 (2011).

S7 S. Felten, C. Q. He, M. Weisel, M. Shevlin, and M. H. Emmert, “Accessing Diverse Azole Carboxylic Acid Building Blocks via Mild C–H Carboxylation: Parallel, One-Pot Amide Couplings and Machine-Learning-Guided Substrate Scope Design”, Journal of the American Chemical Society 144, Publisher: American Chemical Society, 23115–23126 (2022).
